# Flaccid Brachial Monoplegia As Initial Presentation in a Patient With Progressive Multifocal Leukoencephalopathy

**DOI:** 10.7759/cureus.24211

**Published:** 2022-04-17

**Authors:** Lisa B Shields, Vasudeva G Iyer, Hilary A Highfield, Yi Ping Zhang, Christopher B Shields

**Affiliations:** 1 Neurological Surgery, Norton Neuroscience Institute, Norton Healthcare, Louisville, USA; 2 Neurology, Neurodiagnostic Center of Louisville, Louisville, USA; 3 Pathology, Clinical Pathology Accreditation (CPA) Laboratory, Norton Healthcare, Louisville, USA

**Keywords:** pathology, neurosurgery, brain biopsy, nerve conduction study, electromyography, progressive multifocal leukoencephalopathy, neurology

## Abstract

Progressive multifocal leukoencephalopathy (PML) is a demyelinating disease caused by activation of John Cunningham virus (JCV) replication in the setting of impaired cellular immunity. A positive polymerase chain reaction (PCR) assay for JCV DNA in the cerebrospinal fluid (CSF) in conjunction with clinical findings and neuroimaging are diagnostic of PML. A false negative JCV PCR in the CSF may occur, necessitating PML confirmation by brain biopsy. We describe the unique clinical profile of a patient with no prior history of immunocompromise, referred to us for electrodiagnostic evaluation, who initially presented with rapidly progressive weakness of the right upper extremity. The unusual pattern of motor weakness suggested a conduction block or disconnection at the subcortical level. The patient was later diagnosed with atypical small cell lymphocytic lymphoma although not treated with monoclonal antibodies or other forms of chemotherapy. The CSF was negative for JCV, and PML was subsequently confirmed by brain biopsy. This case illustrates an uncommon presentation of PML and highlights the need for a high index of suspicion to diagnose PML.

## Introduction

Progressive multifocal leukoencephalopathy (PML) is a demyelinating disease caused by the reactivation of the polyomavirus John Cunningham (JC) virus (JCV) that causes lytic infection of glial cells [1-7]. PML most often occurs in patients who are immunosuppressed due to HIV, hematologic malignancies, solid organ transplants, or receive monoclonal antibodies for myriad conditions (natalizumab [multiple sclerosis (MS), Crohn’s], efalizumab [psoriasis], rituximab [systemic lupus erythematosus (SLE)], and alemtuzumab [MS]) [1,3-6,8,9]. PML is highly fatal, with a median survival of fewer than three months in patients without AIDS [3].

While JCV-specific antibodies are present in 50%-90% of the adult population worldwide, PML is an exceedingly rare complication with an incidence of 0.2-4.4 cases per 100,000 individuals in the general population [1,6,9,10]. Asymptomatic primary infection with JCV occurs in childhood and may subsequently reactivate from sites of latency and undergo sequential genomic rearrangements, spreading to the central nervous system in immunocompromised individuals [2,5,8,9]. The American Academy of Neurology (AAN) Neuroinfectious Disease Section developed the criteria for the diagnosis of PML which may be confirmed by either (1) the typical neuropathologic histopathologic triad of demyelination, bizarre astrocytes, and enlarged oligodendroglial nuclei as well as the presence of JCV or (2) clinical and radiographic findings consistent with PML and not better explained by other disorders as well as the JCV by polymerase chain reaction (PCR) in the cerebrospinal fluid (CSF) [1,6,9-11].

Presenting symptoms often comprise progressive weakness, sensory deficit, hemianopsia, cognitive dysfunction, aphasia, encephalopathy, incoordination, personality changes, and gait abnormalities [6,8-10]. Characteristic brain MRI findings include hyperintense lesions in the white matter without mass effect or edema on T2-weighted and fluid-attenuated inversion recovery (FLAIR) images which are hypointense on T1-weighted images [3,6,9,10]. Lesions may also be located in the myelinated gray matter such as the basal ganglia or thalamus. CSF evaluation often reveals JCV and excludes diagnoses such as other infections or tumors [10,12]. Few cases have been reported of JCV-negative CSF with subsequent PML confirmation requiring brain biopsy or autopsy [1,3,5,9,11,13,14]. Common brain biopsy findings of PML include the presence of oligodendrocytes with enlarged nuclei and atypical astrocytes, with the immunohistochemistry revealing positive reactivity for JCV-related antigens on abnormal glial cells [3,14]. Electrodiagnostic (EDX) studies have not previously been reported in the evaluation of PML.

The diagnosis of PML may prove challenging despite a high index of suspicion and extensive imaging [5]. We present the unique case of PML with JCV-negative CSF whose PML was later confirmed by brain biopsy. The patient was referred to our center for an EMG study of the unexplained weakness of the right upper extremity. The physical examination and EDX findings are discussed along with the diagnostic challenges. The phenomenon of JCV-negative CSF with PML detection by brain biopsy, differential diagnosis, the proposed mechanism of weakness in PML, and management of PML is also highlighted.

## Case presentation

History, radiological imaging, and physical examination

A 71-year-old male (BMI: 27.12 kg/m^2^) reported the subacute onset of weakness of the right upper extremity as well as swelling and an inability to use the right hand, without pain or paresthesia of the hand. A cerebrovascular accident was initially suspected. A brain MRI with and without Gadolinium contrast revealed infarctions in the left frontal (2.0 x 1.4 cm) and right parietal (2.4 x 2.1 cm) lobes without hemorrhage or significant mass effect. The patient underwent physical therapy without improvement. He was seen by a hand surgeon, who requested electromyography (EMG).

Muscle strength was zero in the finger flexors, finger extensors, wrist flexors, and wrist extensors of the right hand. There was a weakness of pronation and supination of the right forearm. Mild weakness of elbow flexion and extension was noted. Muscle tone was less on the right. Biceps, brachioradialis, and triceps reflexes were symmetrical. The finger flexor reflex was slightly brisker on the right, but Hoffmann’s and Tromner’s signs were negative. Lower extremity reflexes were normal. Sensory examination was normal.

EMG/NCV of the arms

The EDX studies showed conduction abnormalities in the right median and ulnar nerves consistent with entrapment at the wrist and the elbow, respectively. Needle EMG showed a few fibrillations and positive waves in the first dorsal interosseous (FDI), abductor digiti minimi (ADM), and abductor pollicis Brevis (APB), flexor pollicis longus (FPL), and extensor indicis (EI) muscles. The patient was unable to recruit even a single motor unit in these muscles; a few motor units of normal morphology were recruited in the pronator teres (PT), brachioradialis, and triceps muscles. Normal motor unit recruitment was noted in the biceps and deltoid muscles.

Accurate localization was elusive on the basis of clinical and EDX findings. While the pattern of muscle weakness suggested an upper motor neuron lesion, the muscles were flaccid rather than spastic. The abnormal conduction in the median nerve at the wrist and ulnar nerve at the elbow was consistent with entrapment neuropathy but were likely incidental findings. Two possibilities were considered: (1) Selective involvement of the motor fibers at the level of the brachial plexus or cervical nerve roots (lack of sensory symptoms and signs made this diagnosis less likely) and (2) a cortical lesion involving the motor area (absence of speech involvement and focal seizures made this diagnosis less likely). The total inability to recruit any motor units in the distal muscles was so striking that the possibility of conversion disorder was in the differential diagnosis initially. The presence of fibrillation was difficult to explain with an upper motor neuron disorder although there has been one report of such an occurrence [15].

MRIs of the cervical spine and brachial plexus showed extensive lymphadenopathy, and the possibility of compression of the brachial plexus by enlarged lymph nodes was considered. Within 4-5 days the muscle weakness had involved the entire right upper extremity including shoulder girdle muscles with the pattern of complete absence of motor units on attempted voluntary contraction. No sensory abnormalities were present. No speech or cognition problems were noted. Over the next few days, he developed hyperreflexia of the deep tendon reflexes on the right, favoring an upper motor neuron lesion. Within a week, he developed weakness of the lower extremities causing ambulatory difficulty and slurred speech.

Hospital course

**Figure 1:**
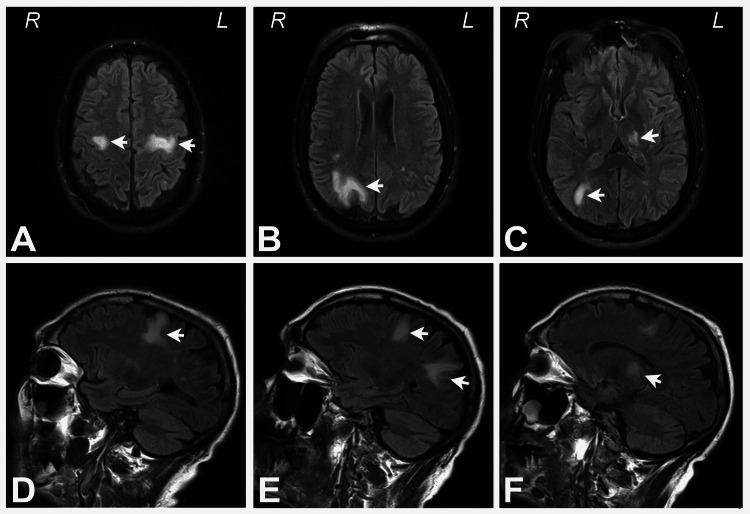
Unenhanced FLAIR axial and sagittal images demonstrating bifrontal, right parietal, and bilateral thalamic lesions Top row: Unenhanced FLAIR axial images demonstrating the (A) bifrontal, (B) right parietal, and (C) left thalamic and right thalamic lesions (arrows). Bottom row: Unenhanced FLAIR sagittal images showing the (D) left posterior frontal, (E) right posterior frontal and right parietal, and (F) left thalamic lesions (arrows).

**Figure 2:**
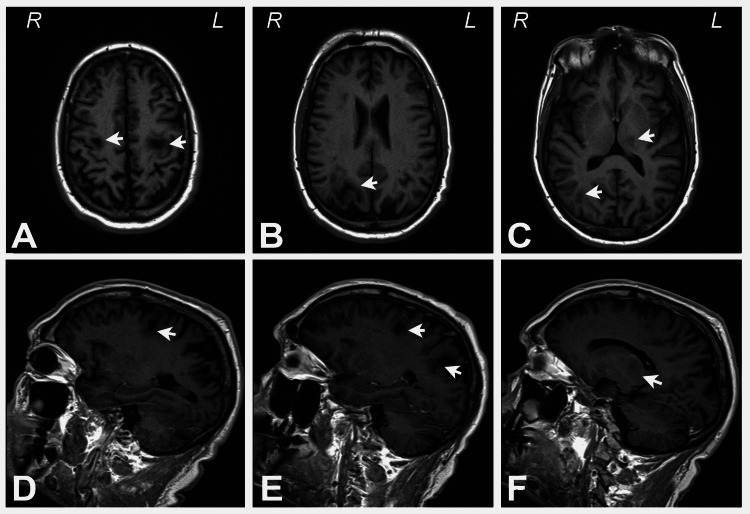
Unenhanced T1 FLAIR axial and sagittal images demonstrating the bifrontal, parietal, and bilateral thalamic lesions Top row: Unenhanced T1 FLAIR axial images demonstrating the (A) bifrontal, (B) right parietal, and (C) left thalamic and right thalamic lesions (arrows). Bottom row: Unenhanced T1 FLAIR sagittal images showing the (D) left posterior frontal, (E) right posterior frontal and right parietal, and (F) left thalamic lesions (arrows).

A repeat brain MRI with and without Gadolinium contrast 6 weeks after the first one demonstrated new and progressive foci of abnormal signal intensity and enhancement within the bifrontal lobes, right parietal lobe, and bilateral thalamus (Figures 1A-1F, 2A-2F).

Foci of abnormal signal intensity developed within the lateral left basal ganglia. The differential diagnosis included a demyelinating or inflammatory process or subacute ischemia. A PET/CT scan revealed extensive hypermetabolic adenopathy throughout multiple lymph node chains and moderate splenic enlargement, suggestive of lymphoma or leukemia. A left axillary node biopsy stained positive with CD45, CD20, CD23, and ZAP-70 with 40% of the tumor nuclei in the proliferation center staining positive with Ki-67. Flow cytometry was negative for CD5 (typically seen in classical small cell lymphocytic lymphoma), and fluorescence in situ hybridization (FISH) testing revealed trisomy 12. The diagnosis was atypical small cell lymphocytic lymphoma. CSF analysis is presented in Table 1.

**Table 1:** Cerebrospinal fluid analysis of progressive multifocal leukoencephalopathy in our case JC virus: John Cunningham virus, PCR: polymerase chain reaction, B. Burgdorferi: Borrelia Burgdorferi, IgG: Immunoglobulin G, IgM: Immunoglobulin M, VDRL: venereal disease research laboratory, HIV-1 and HIV-2: human immunodeficiency virus-1 and -2, CSF: cerebrospinal fluid, WBC: white blood cells

Cerebrospinal Fluid	Laboratory Value	Reference Range
JC virus PCR quantitative	Not detected	<100 COPYS/ML
West Nile virus	Negative	
B. Burgdorferi IgG	Negative	
B. Burgdorferi IgM	Negative	
VDRL and VDRL titer	Non-reactive	
Lyme disease antibodies	Negative	
HIV-1 and HIV-2 antibody/antigen combination	Non-reactive	
Total protein	79 mg/dl	12-60 mg/dl
CSF culture	No growth	
Gram stain	No organisms seen, no WBCs	
India ink prep	Negative for encapsulated yeast	
Oligoclonal bands detected in CSF	IgG: 6.8 mg/dl	0-3.4 mg/dl
	Albumin: 42.2 mg/dl	13.9-24.6 mg/dl
	IgG synthesis rate: 5.8 mg/24 hr	0-3.2 mg/24 hr

**Figure 3:**
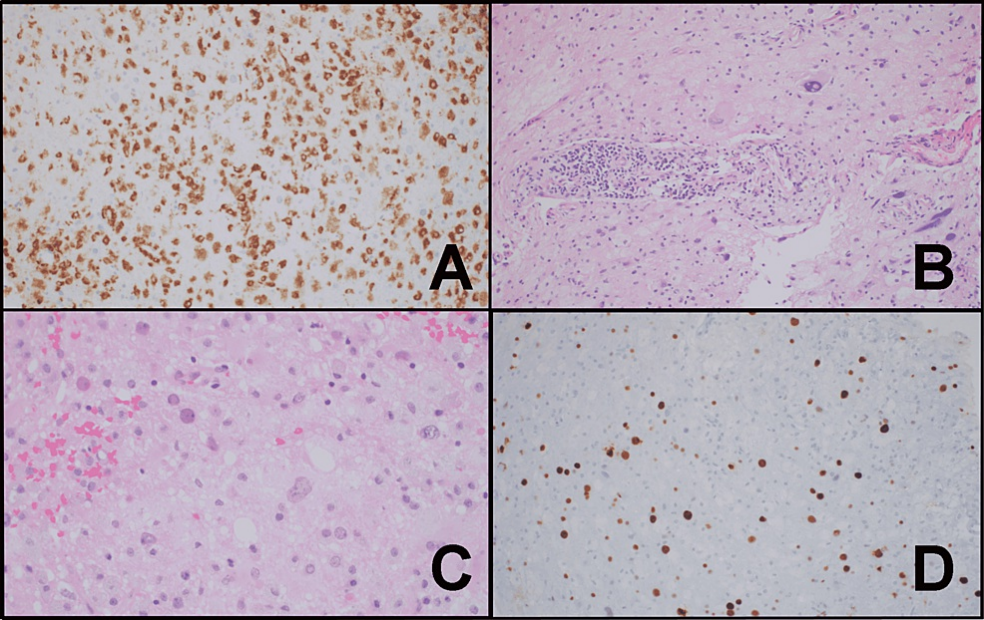
Microscopic examination of a right parietal brain stereotactic biopsy Microscopic examination of the right parietal brain stereotactic biopsy revealed (A) the macrophage infiltrate by CD68 immunohistochemistry (H&E, 200x); (B) perivascular chronic inflammation and bizarre astrocytes; (C) oligodendrocytes with glassy nuclei (H&E, 400x); and (D) intranuclear viral inclusions by SV40 immunohistochemistry (H&E, 200x).

Microscopic examination of a right parietal brain stereotactic biopsy showed dense macrophage infiltration (Figure 3A).

There was marked reactive gliosis including large astrocytes with bizarre morphology (Figure 3B). There was perivascular lymphocytic cuffing, but no evidence of an abnormal lymphoid population (Figure 3B). Oligodendroglia had enlarged nuclei and prominent intranuclear inclusions (Figure 3C). Immunohistochemical stains demonstrated the extent of reactive gliosis (GFAP) and macrophage infiltration (CD68, Figure 3C). The background lymphocytes were mature appearing T-cells (CD3) rather than B-cells (CD20). The Epstein-Barr encoding region (EBER) was negative. Flow cytometry immunophenotyping (using eight antibodies CD3, CD4, CD8, CD19, CD20, CD45, Kappa Surface Light Chains, Lambda Surface Light Chains) of cells obtained from the right parietal lesion failed to reveal a monoclonal B-cell population. The SV40 immunohistochemical stain was strongly positive within the enlarged oligodendroglial nuclei (Figure 3D). Immunohistochemistry for p53 and Ki-67 showed a similar pattern of positivity. GFAP and vimentin confirmed the extent of reactive gliosis. Silver staining (Bielschowsky) demonstrated overall preservation of axons, characteristic of a demyelinating process. Luxol fast blue also confirmed the presence of myelin debris within the macrophage cytoplasm, a finding typical of demyelinating lesions. The brain biopsy findings were diagnostic of PML.

There was a steady progression of muscle weakness with more severe involvement of the lower extremities and the appearance of ptosis. Oncology recommended rehabilitation prior to starting chemotherapy for the lymphoma. Within two and a half months of symptom onset, the patient developed metabolic encephalopathy, fever, profound weakness, and was unresponsive. He died one week later after experiencing sepsis.

## Discussion

There are several unique features in the clinical picture of this patient. The onset of PML in immunocompetent patients is extraordinarily rare, as reflected by only a few reported cases [7,16-19]. In Tan and colleagues’ case of PML diagnosed in an immunocompetent patient, the presenting symptoms progressed over a two-year period instead of a few months, and the brain MRI revealed extensive T2/fluid-attenuated inversion recovery hyperintensities of white matter lesions that suggested a cellular immune response [7]. These authors proposed that their patient had experienced a transient dysfunction of cellular immunity such as subclinical parvovirus B19 infection that was sufficient to promote reactivation of JCV [7]. Our case highlights the diagnosis of PML in a patient without AIDS or a previous exposure to monoclonal antibodies or other forms of chemotherapy. He was not known to be immunocompromised at the initial presentation. A subsequent MRI showed lymphadenopathy, which led to a biopsy and discovery of small cell lymphocytic lymphoma later in the course of PML.

Similar to our patient, only six case reports have been published with negative JCV DNA in the CSF with subsequent confirmation of PML by either brain biopsy or autopsy (Table 2) [1,3,5,9,13,14].

**Table 2:** Progressive multifocal leukoencephalopathy with negative JC virus DNA in the cerebrospinal fluid MS: multiple sclerosis, JCV: JC virus, RRMS: relapsing-remitting multiple sclerosis, PML-IRIS: progressive multifocal leukoencephalopathy immune reconstitution inflammatory syndrome, IVIG: intravenous immune globin, RA: rheumatoid arthritis, SLE: systemic lupus erythematosus, DLE: discoid lupus erythematosis, PCR: polymerase chain reaction

Study	Age/ Gender	Presenting Symptoms	Concurrent Medical Conditions	Brain MRI	Brain Biopsy	Outcome
Babi et al. [1]	75F	Left hemiplegia, global decline	RA treated with methotrexate, adalimumab	Right subcortical T2-FLAIR hyperintense lesion, left hemispheric periventricular white matter	Not performed	No treatment; died 3 months after symptom onset; autopsy confirmed PML and JCV by immunohistochemistry
Ikeda et al. [3]	32F	Right hemiplegia, motor aphasia	SLE treated with prednisolone, cyclophosphamide, tacrolimus	Large white matter lesion in left frontal lobe, small dot-like lesions in both occipital lobes	Oligodendrocytes with enlarged nuclei and atypical astrocytes; immunohistochemistry revealed positive reactivity for JCV-related antigens on abnormal glial cells; JCV DNA detected by PCR of fresh-frozen brain tissue	Treated with mirtazapine; requires minimal aid in daily life although declined mental activity 24 months after symptom onset
Kuhle et al. [5]	48F	Left hypoesthesia, dysesthesia, equilibrium disturbance, left leg weakness, unsteady gait	RRMS treated with natalizumab	Hyperintense, contrast-enhancing, subcortical, lesion in right central region	qPCR positive for JCV; JCV brain tissue viral load positive; direct sequencing revealed rearranged noncoding control region JCV variant with partial deletions and duplication	PML-IRIS developed, treated with IVIG; clinical status returned close to baseline 22 months after symptom onset
Inamullah et al. [9]	65M	Right-sided weakness, facial weakness, cognitive decline	Dermatologic sarcoidosis treated with hydroxychloroquine	Enlargement of non-enhancing subcortical and periventricular T2 hyperintensities with Wallerian degeneration	Extensive gliosis, abundant lipid-laden macrophages, large ground glass viral inclusions; SV40 immunoreactive in cells with viral cytopathologic changes conforming PML	No treatment; died 8 months after symptom onset
Landry et al. [13]	31F	Clumsiness of left hand, difficulty walking	MS treated with corticosteroids, plasma exchange	“Consistent with given diagnosis of MS, but pattern of white matter changes was not the most typical”	Demyelination, myelin-debris-laden foamy macrophages, enlarged nuclei; in situ hybridization confirmed JCV DNA	No treatment; died 5 months after symptom onset
Villani et al. [14]	58M	Encephalopathy, incoordination	DLE, no treatment	T2/FLAIR signal hyperintensities in bilateral parietal and temporal subcortical white matter and subcortical U-fibers, hypointense on T2-weighted sequences and without contrast	Oligodendrocytes with enlarged nuclei and intranuclear inclusion, positive for polyomavirus SV40 antigen	Treated with plasma exchange before PML diagnosis; died 3 weeks after brain biopsy without treatment
Present case 2022	71M	Right arm weak, inability to use right hand	Atypical small cell lymphocytic lymphoma diagnosed after presentation, no treatment	Infarctions left frontal, right parietal; progressive foci of abnormal signal intensity and enhancement within posterior frontal, right parietal, left thalamus, lateral left basal ganglia	Macrophage infiltrate with reactive gliosis, large astrocytes with bizarre morphology; oligodendroglia with enlarged nuclei and prominent intranuclear inclusions; JCV strongly positive within enlarged oligodendroglial nuclei	No treatment; progressive weakness of right arm, right leg, right chest, and ptosis; died 2 ½ months after symptom onset

Two patients were diagnosed with MS, and one each with RA, SLE, dermatologic sarcoidosis, and discoid lupus. Five patients had been treated with monoclonal antibodies or immunosuppressive medications appropriate for their medical conditions. These patients presented with classic features of arm/leg weakness, gait abnormalities, cognitive decline, incoordination, and encephalopathy. Brain MRI findings consisting of white matter changes and T2-FLAIR hyperintense lesions were noted. Five patients underwent a brain biopsy that confirmed PML by observing demyelination, oligodendrocytes with enlarged nuclei, atypical astrocytes, extensive gliosis, and/or JCV DNA. Immunohistochemistry performed at autopsy confirmed JCV (PML) in one patient. Four patients did not undergo treatment following the PML diagnosis and died between three and eight months after symptom onset. One received mirtazapine and attained improvement apart from decreased mental activity 24 months after symptom onset. Another patient was treated with IVIG for PML-immune reconstitution inflammatory syndrome (PML-IRIS) with a near return to baseline 22 months after symptom onset.

In Wijburg and colleagues’ study of 56 individuals with MS and natalizumab-associated PML, nine (16.5%) had undetectable JCV DNA by PCR in the CSF [11]. Patients with a positive PCR had larger total PML lesion volumes than those with undetectable JCV DNA in the CSF (median volume: 22.9 mL vs 6.7 mL). Additionally, the PML lesion volume was higher in patients with PML symptoms and those with more widespread lesion dissemination.

CSF PCR for JCV is highly specific (92%-99%) and sensitive (74%-93%); however, false negatives may occur [1,3,5]. Myriad reasons may account for JCV-negative CSF, including early stages of PML, inadequate choice of JCV DNA PCR targets, low titers of JCV DNA in the CSF, using different detection thresholds, low volume of the specimen, loss of DNA during concentration, and lack of spontaneous dissemination into the subarachnoid space [1,3,5,20]. Interestingly, repeat JCV testing of the same CSF targeting a different region of the genome may yield a positive result [13]. Several diagnostic approaches have been developed to ascertain the presence of JCV in patients with JCV-negative CSF but have suspected PML based on clinical symptoms and brain MRI findings. Assessment of intrathecal antibody synthesis to JCV by determining the CSF JCV antibody index as well as more ultrasensitive PCR assays may improve diagnostic accuracy [1,5,11]. A brain biopsy is a gold standard for conclusively confirming PML if repeat CSF examinations are negative and the symptoms strongly suggest PML. It has been proposed that an early brain biopsy may be essential for making a timely diagnosis of PML since the detection of JCV DNA in the CSF is not always reliable [3]. Numerous disorders should also be considered in the differential diagnosis of PML, including autoimmune and inflammatory conditions (MS and acute disseminated encephalomyelitis), infectious conditions such as HIV encephalitis, and varicella-zoster virus encephalopathy, mitochondrial encephalopathies, CNS vasculitis, and reversible posterior leukoencephalopathy [1].

Another unique feature of the present case is the circuitous route it took to make the final diagnosis of PML, involving a hand surgeon, neurologist/electromyographer, neurosurgeon, and neuropathologist in tandem. The pattern of motor weakness was unusual and posed a diagnostic dilemma to localize the lesion site. The initial presentation was flaccid brachial monoplegia affecting the distal muscles. There was not even a flicker of muscle contraction, a most unusual pattern, probably suggestive of “disconnection” of the premotor/motor cortex. We propose that the pathway between the premotor/supplementary motor and the motor strip may have been blocked by demyelination from PML. The patient’s signs and symptoms progressed over a two-month period in a series of three stages presumably concurrent with progressive damage to the white matter. In the first stage, the patient had some motor function but no volitional motor activity globally in the distal muscles. During this stage, the white matter connections between the prefrontal lobe and the motor cortex (posterior frontal lobe) may have been damaged by PML. During the second stage, the patient experienced progressive motor paralysis of the right arm and right leg, at which time white matter damage progressed posteriorly affecting the connections in the motor strip. In the final stage, demyelination may have become much more widespread also involving the brainstem.

As no antiviral agent currently exists against JCV, immune reconstitution is the goal in treating and prolonging survival in PML which is most successful with early diagnosis and limited disease progression [2,3,5,6,8,11]. Treatment aimed at restoring immune function includes removing the offending medication (if it was the cause of PML), plasmapheresis, and starting mirtazapine (blocks viral entry into uninvolved glial cells), mefloquine (inhibits JCV proliferation in vitro), maraviroc, cytarabine, acyclovir, cidofovir, immunoglobulin, anti-PD1-antibodies (pembrolizumab and nivolumab), or immunomodulatory agents (IL-2, IL-7, and VP1-antigen immunization) [7-9]. Based on the literature to date, therapy with allogeneic T cells provides the most promising results in the treatment of PML [20]. Long-term survivors often face significant morbidity due to persistent low-level viral replication, with 70% suffering residual neurological disability and 44% experiencing seizures [2].

## Conclusions

Physicians should have a high index of suspicion of PML in patients who display progressive weakness of the upper extremity. One should also be aware that PML can very rarely occur in immunocompetent individuals. A prompt brain biopsy is mandatory to confirm the diagnosis in patients whose clinical picture and MRI suggest PML and in whom CSF is negative for JCV.
